# N-acetyl-L-cysteine and lauric acid; effective antioxidant and antimicrobial feed additives for juvenile Pacific white shrimp (*Litopenaeus vannamei*) cultured at high stocking density

**DOI:** 10.1371/journal.pone.0315819

**Published:** 2025-01-15

**Authors:** Shafaq Fatima

**Affiliations:** Department of Biological Sciences, Purdue University Fort Wayne, Fort Wayne, Indiana, United States of America; Kafrelsheikh University, EGYPT

## Abstract

Present study aimed at improving the immune and antioxidant response of Pacific white shrimp (*Litopenaeus vannamei*) cultured at high stocking density fed with 0.2% supplementation of lauric acid (LA) and N-acetyl-L-cysteine (NAC). Shrimp (initial average weight = 0.65 g; n = 270) were grown at low stocking density (LSD) (n = 10/0.80 ft^3^ per replicate) and high stocking density (HSD) (n = 20/0.80 ft^3^ per replicate). They were randomly distributed into five groups (T1: negative control at LSD, T2: positive control at HSD, T3: at HSD and fed with LA supplement diet, T4: at HSD and fed with NAC supplemented diet, T5: at HSD and fed with combination of LA and NAC). All these five treatments were studied in triplicates and study continued for eight weeks. Better growth and higher levels of glucose, total protein, total hemocyte count and phagocytic index were observed in shrimp fed with NAC and LA supplemented diets. Observed survival rate and feed conversion ratio in all treatments was 75–89% and < 0.82, respectively. All parameters indicating stress were observed to be higher in T1 as compared to T2. Improved expression of superoxide dismutase and glutathione peroxidase and lower levels of malondialdehyde genes in T3, T4 and T5 showed that supplementation with these nutraceuticals can improve antioxidant response at high stocking density. A parallel increase was observed in the profiles of prophenoloxidase and lysozyme, underscoring the immune-boosting effects of both NAC and LA. This finding was further supported by higher expression of innate immune signaling pathway-related gene, toll like receptor-2 in T3, T4 and T5. In conclusion, NAC and LA, can possibly improve the resistance of white pacific shrimp against oxidative stress and pathogens when cultured in intensive production system.

## 1. Introduction

Global shrimp farming is mainly concentrated in Asia and Americas while Asian countries and Latin American nations contribute to 83.4% and 16.3%, respectively of shrimp production. The primary species involved are the Pacific white shrimp (*Litopenaeus vannamei*) with 83% of the production and the tiger prawn (*Penaeus monodon*) with 12% [1]. Commercial shrimp farming has adopted a variety of technologies and production systems (lined ponds, raceways, tanks, biofloc) to fulfill 94% of production output from only 58% of globally available production area of 2.4 million hectares [2]. Shrimp aquaculture is presently facing a range of challenges that are affecting its worldwide expansion and stability. These challenges encompass feed expenses, market fluctuations, diseases, and the quality of broodstock [3]. Among all cultured species, shrimp farming has been most significantly affected by infectious diseases. A highly developed immune system is crucial for preventing disease outbreaks in intensive culture and minimizing the reliance on antibiotics. Several categories of feed additives (amino acids, minerals, probiotics, prebiotics, peptides, organic acids, nucleotides) have been used in aquaculture to improve the innate and adaptive immunity against common pathogens and oxidative stress [4].

Among innovative immunomodulatory factors, lauric acid, a saturated, medium-chain fatty acid, naturally found in high concentrations in oils (coconut, palm, black soldier fly larvae) has secured its place in aquaculture. Lauric acid exhibits strong antibacterial and antiviral properties, particularly effective against gram-positive bacteria [5], inhibits biofilm formation, membrane biosynthesis [6] and virulence factors [7], may eliminate vegetative cells and spores [8] and improve antioxidative capacity [9]. This feed additive induces dimerization of Toll-like recptor-2 (TLR-2) with TLR-1 or TLR-6 leading to the activation of MyD88-dependent signaling pathways and subsequent production of proinflammatory cytokines in an NFκB-dependent manner [10]. TLR-2 expressed in innate immune cells, recognizes a variety of pathogen-associated molecular patterns (PAMPs) (lipopeptides, peptidoglycan, and lipoteichoic acids, zymosan, mannan, tGPI-mucin) derived from gram-positive bacteria [10] Above mentioned role of purified lauric acid as feed additive has been reported in broiler [11], mammals [8] and in black sea bream (*Acanthopagrus schlegelii*) [9]. However, to the best of our knowledge, no study has investigated its role in improving innate immunity in shrimp or other crustacean.

Hypoxia is the key challenge under highly stocked husbandry conditions/intensive culture which compromises immune response of animals and increases their susceptibility to diseases and reduced growth [12]. Stressors like hypoxia can lead to increased generation of reactive oxygen species (ROS), such as hydrogen peroxide (H_2_O_2_), superoxide anion (O_2_• -), and hydroxyl radicals (•OH), in aerobic organisms [13]. Various mechanisms including mitochondrial electron transport chain [14] and NADPH oxidase complex [15], produce excessive ROS under hypoxic stress. These radicals cause peroxidation of polyunsaturated fatty acids (PUFA) and forming lipid hydroperoxide as primary and malondialdehyde (MDA) as secondary product [16]. MDA is widely used convenient biomarker for oxidative stress to determine lipid peroxidation of PUFA especially omega-3 and omega-6 fatty acids. Both animals and plants have integrated antioxidant defense systems comprising of antioxidant enzymes (catalase, superoxide dismutase, glutathione peroxidase) and non-enzymatic antioxidant molecules (glutathione and thioredoxine) to manage cellular ROS balance. Superoxide anion (O_2_• -) are dismutated into H_2_O_2_ and O_2_ by superoxide dismutase (SOD) [12]. Catalysation of this liberated H_2_O_2_ by catalase enzyme converts it in to H_2_O and O_2_. Similarly, glutathione peroxidase reduces H_2_O_2_ to H_2_O by employing glutathione as an electron donor [17].

Artificial antioxidants such as N-acetyl-L-cysteine (NAC) are used as agonist of natural ROS scavengers. NAC serves as a synthetic precursor to intracellular cysteine and glutathione stimulating direct scavenge of free radicals through the redox potential of thiols or enhancing cellular glutathione levels, contributing to its anti-ROS and anti-malondialdehyde activity [2, 12]. It has a confirmed importance as anti-inflammatory and anti-apoptotic agent via the MAPK/NF-κB/Nrf2 and other signaling pathways, protecting cells and organelles against oxidative stress in humans [18] and fish [19]. There is growing evidence that NAC as a feed additive improves antioxidant activity in common carp (*Cyprinus carpio*) [19], tilapia (*Oreochromis niloticus*) [20], rainbow trout (*Oncorhynchus mykiss*) [21], large yellow croaker (*Larimichthys crocea*) [22], clearfin livebearer (*Poeciliopsis lucida*) [23], and Chinese mitten crab (*Eriocheir sinensis*) [24, 25].

Considering the proven role of LA and NAC as antimicrobial and antioxidant agents, present study investigated if they could improv immune and antioxidant response in Pacific white shrimp cultured at high stocking density. The main objective was to find out if these compounds can be good candidates for potential use as feed additives in intensive shrimp farming at commercial scale.

## 2. Materials and methods

### 2.1 Preparation of diets and experimental design

Four different diets were prepared with supplementation of lauric acid (Sigma, W261408) and N-Acetyl-L-cysteine (Sigma, A7250) (D1: Zero supplementation, D2: supplemented with LA, D3: supplemented with NAC, D4: supplemented with LA+NAC). A total of 2 g of LA was dissolved in to 100 ml of ethanol (> 98%) while same quantity of NAC was mixed in 20 ml of deionized water. These doses were selected after Ullah et al. [9] and Wang et al. [25]. The required volume of these solutions was sprayed on 1 Kg of commercial feed (40% CP, 2 mm pellet, Ziegler, USA), thoroughly mixed, left overnight for drying and stored at 4°C. Control diet (D1) was sprayed with equal volume of ethanol and deionized water only.

This study was conducted according to animal ethics protocols of Purdue University Fort Wayne. Shrimp (initial average weight = 0.65 g), procured from School of Fisheries and Aquaculture, Auburn University, USA and acclimatized in 100-gallon tank for two weeks. After acclimatization period, a total of 270 shrimp were randomly distributed in fifteen aquaria (Area = 4.50 ft^3^ Water area = 2.12 ft^3^) to study five different treatments in triplicates (Table 1). These stocking densities were adjusted following the stocking density value in extensive shrimp farming (two shrimp per 135 cm^2^ to obtain biomass of 4.44 kg/meter^2^ at 100% survival). Additional three shrimp were overstocked in each replicate to compensate the expected mortality (n = 45, number not shown in Table 1). Shrimp were fed four times a day. To calculate daily feed ration, target of FCR 1.8 was used until the estimated weight gain of 1 g/week is achieved (Galkanda‐Arachchige et al., 2021). Expected growth rate of 1 g/week was assumed for feed calculation. Daily feed ration was adjusted according to observed mortality and feed consumption. Dead shrimp were immediately removed when observed. Trial continued for eight weeks. At end of the trial, total length and weight of each shrimp in all replicates of each treatment were measured to calculate the mean final weight, weight gain, condition factor and feed conversion ratio (FCR) other than survival rate.


$$\text{Condition}\;\text{Factor}\;(\text{K})=\left[\frac{\text{Weight}}{{\text{Length}}^{3}}\right]\times 100$$

$$\text{Feed}\;\text{Conversion}\;\text{Ratio}\;(\text{FCR})=\frac{\text{Total}\;\text{feed}\;\text{given}\;(\text{Dry}\;\text{weight})}{\text{Total}\;\text{weight}\;\text{gain}\;(\text{Wet}\;\text{weight})}$$

$$\text{Survival}\;\text{Rate}\;(\%)=\left[\frac{\text{Number}\;\text{of}\;\text{surviving}\;\text{animals}}{\text{Initial}\;\text{number}\;\text{of}\;\text{animals}}\right]\times \text{100}$$

**Table 1:** Details of different stocking density groups and their relevant diet, fed during the study period.

Treatment	Stocking Density + Supplementation	n/replicate	N
T1	Low stocking density, Fed with D0	10	30
T2	High stocking density, Fed with D0	20	60
T3	High stocking density, Fed with D1 (LA)	20	60
T4	High stocking density, Fed with D2 (NAC)	20	60
T5	High stocking density, Fed with D3 (LA+NAC)	20	60

Salinity, water temperature, pH, and dissolved oxygen were monitored every day. Ammonia, nitrite, and nitrate were recorded after every four days. Water temperature was maintained within the range of 27–28°C while salinity, average pH and dissolved oxygen were 8 ppm, 8.1, and 77%-82%, respectively throughout the trial. Ammonia, nitrite, and nitrate were found below the detection limit of the testing kit (API, USA) throughout the trial.

### 2.2 Hemolymph and tissue sampling

Shrimp were randomly collected from each replicate (n = 10/replicate) and euthanized by using ice cold water. Hemolymph was extracted from the hemolymph sinus at the base of each walking leg using 1 mL syringe tipped with a 26-gauge needle. Two sub samples of hemolymph were collected from each treatment. One sub sample (A) was collected without anticoagulant while the second (B) was mixed with equal volume of anticoagulant. The total volume of hemolymph was 100 μL and 50 μL in sub sample A and B, respectively. The anticoagulant solution [26] was prepared to contain 30 mM sodium citrate tribasic dihydrate (Sigma S4641); 0.34 M sodium chloride; 10 mM ethylene diamine tetra acetic acid (EDTA) (Sigma, E9884); in de-ionized water (pH: 7.5). Sub sample A was used to determine glucose, and total protein immediately while sub sample B was used to count total circulating hemocytes/ml. One set of sub sample A from all treatments was stored in liquid nitrogen to measure malondialdehyde (MDA), expression of prophenoloxidase (PPOD) and toll-like receptor-2 (TLR-2) genes. Hepatopancreas were dissected and immediately stored in liquid nitrogen to determine the expression of lysozyme (LYS), superoxide dismutase (SOD), and glutathione peroxidase (GPX) genes. Hemolymph and hepatopancreas were stored at -80°C prior to RNA extraction. Two drops (100 μl) of sampled hemolymph (with anticoagulant) were added on to the glass slide and incubated for 90 minutes at room temperature. Zymosan solution was prepared by mixing 100 mg of Zymosan with 10 ml of deionized water. Zymosan solution was (100 μl) to each drop of cell suspensions and incubated for additional 60 minutes at room temperature. These slides were washed with PBS, dried and stained with Wright Giemsa. Slides were microscopically studied using Leica optical microscope (BM-700, USA) at 200 X and 400 X to determine the phagocytic index following the given formula.


$$\text{Phagocytic}\;\text{Index}\;(\text{PI})=\frac{\text{Bacteria}\;\text{phagocytized}\;\text{by}\;\text{phagocytic}\;\text{haemocytes}}{\text{Phagocytic}\;\text{haemocytes}}$$

### 2.3 RNA Isolation, cDNA Synthesis and qPCR

Expression of SOD, GPX, and LYS were measured in hemolymph while TLR-2 and PPOD was determined in hepatopancreas tissues. Housekeeping gene (β-Actin) was measured both in hemolymph and hepatopancreas. Total RNA was extracted from hepatopancreas and hemolymph samples using NEB Monarch RNA Isolation Kit with DNAse digestion (Spin-Column based method). Nearly, 20–30 mg of these tissues were used for extraction. These samples were homogenized in 300 μL of DNA protection reagent using Dounce homogenizer (ThermoFisher, USA). The ratios of A260/A230 and A260/A280 were measured in extracted RNA samples using Nanodrop spectrophotometer (NanoDrop One, Thermoscientific, USA). The results showed that the extracted RNA samples were pure (A260/A230 > 2.0; A260/A280 > 1.8) (11). cDNA was generated using the ThermoFisher Verso cDNA Synthesis Kit with Oligo-dT primers and stored at -80°C until qPCR was performed.

Specific primer pairs used for gene analysis and their sources are given in Table 2. All primers were synthesized by Integrated DNA Technologies, USA. The real-time quantitative PCR (RT-qPCR) was performed in Bio-Rad Real-Time Thermocycler (CFX Connect, Singapore) using iTaq Universal SYBR® Green Supermix (Bio-Rad, USA) with the cDNA. Reactions consisted of final concentrations of 1/20 volume (1μL) of cDNA, 10 μmol per primer (1μL/primer), iTaq Sybr Green (10μL) and nuclease free H_2_O (7μL) in a final volume of 20μL. The conditions for the RT-qPCR were as follows: Initial denaturation at 95°C for 2 min, followed by 40 cycles (95°C for 5 sec, 60°C for 30 sec). Each sample was run in triplicate with the β-actin gene as an internal control. Non template control reactions were included for every primer set by replacing template cDNA with nuclease free H_2_O. cDNA dilution of 1:20 was used for β-actin, SOD and GPX. However, undiluted cDNA was used for LYS, TLR-2 and PPOD as Ct values for these genes were below detection at 20 times cDNA dilution. The real-time standard curves of β -actin, SOD and GPX were prepared using specific cDNA as a template. Delta-Delta Ct method was used for data analysis of LYS, TLR-2 and PPOD.

**Table 2:** Specific primers for control and target genes were selected from different studies.

Gene name	Sequence of primers (5′−3′)	Source
B-Actin	F: CGAGGTATCCTCACCCTGA R: CGGAGCTCGTTGTAGAAGG	[27]
Superoxide Dismutase	F: TTAGTGGGACCTCGTACGGT R: CTCAAGCGTGACCTATGACC	[28]
Glutathione Peroxidase	F: AGTCGATGTCAACGGGTCAAC R: GCTGAACCTCTTAAACGGCTG	[27]
Toll Like Receptor-2	F: CATGCCTGCAGGACTGTTTA	[22]
	R: GGCCTGAGGGTAAGGTCTTC	
Prophenoloxidase	F: GCCTTGGCAACGCTTTCA R: CGCGCATCAGTTCAGTTTGT	[29]
Lysozyme	F: GCAAGAACGTCTGCAAAATCC R: -CCAGCACTCTGCCATGTACTG	[30]

F: forward, R: reverse

### 2.4 Determination of malondialdehyde in hemolymph

Hemolymph samples from each treatment were homogenized in nine volumes of normal saline by and then centrifuged at 3000 rpm/min for 20 min at 4°C. The supernatant was removed and used for ELISA. For malondialdehyde (MDA) (nmol/ml), commercial kit (MY Biosource, USA, CAT No. MBS1601664) with an assay range of 0.05 ng/ml– 30 nmol/ml was used. Intra-assay and inter-assay precision was <8% and <10%, respectively. Sensitivity of assay was 0.025nmol/ml.

### 2.5 Statistical analysis

The mean ± standard deviation (S.D) was used to express the results. To find significant differences between groups, statistical analysis was performed using one-way analysis of variance (ANOVA) with a significance level set at P< 0.05. The variation between means was examined further using the Tukey’s post-hoc test after the normality and homogeneity of variances were assessed using the Kolmogorov-Smirnov test and the Levene test, respectively. The parameters that showed substantial variation after the DMRT test were indicated by superscripts. SPSS (IBM Corp., Armonk, New York), version 20 was used for all analyses.

## 3. Results

### 3.1 Growth, glucose, total protein, total hemocyte count and phagocytic index

Higher values of weight gain were observed in T3, T4 and T5 while maximum value was noted in T4 (Table 3). Values of condition factor showed a significant difference (p<0.05) and noted to be less than 1.00 in all treatments. FCR was also calculated to be less than 1.00 in all treatments. The levels of total protein, glucose, total hemocyte count and phagocytic index were noted to be significantly different (p<0.05) among all five treatments but quantitatively higher in T3, T4 and T5 as compared to others. The highest values of these parameters were observed in T4.

**Table 3:** Details of different growth parameters, glucose, total protein, total hemocytes count and phagocytic index at end of trial. Subsets were calculated by using the Tukey’s post-hoc test in One-Way ANOVA. They show the variance between five treatments along the column.

Tr.	TBW (g)	Weight Gain (g)	K (%)	Survival (%)	FCR	Glucose (mg/dl)	TP (g/100 ml)	THC	PI
T1	1.31 ± 0.26^a^	0.96	0.59 ± 0.06^bc^	88	0.93	43.50 ± 4.76^b^	6.77 ± 0.36^a^	1.39 × 10^6 a^	0.24 ± 0.06^a^
T2	1.64 ± 0.26^a^	0.99	0.50 ± 0.02^a^	75	0.91	40.50 ± 3.21^a^	8.48 ± 0.78^b^	1.40 × 10^6 a^	0.37 ± 0.01^b^
T3	2.50 ± 0.45^c^	1.85	0.60 ± 0.05^b^	85	0.85	51.75 ± 2.36^c^	13.62 ± 0.37^c^	3.71 × 10^6 b^	2.48 ± 0.46^c^
T4	2.60 ± 0.31^c^	1.95	0.68 ± 0.03^c^	89	0.82	55.50 ± 2.00^d^	17.13 ± 0.50^d^	3.84 × 10^6 b^	2.63 ± 0.52^c^
T5	2.30 ± 0.40^b^	1.64	0.57 ± 0.02^b^	86	0.84	52.00 ± 4.38^c^	14.26 ± 0.77^c^	3.37 × 10^6 b^	3.31 ± 0.64^d^

Tr: treatment, TBW: total body weight, TP: total protein, THC: total hemocytes count, FCR: feed conversion ratio, PI: phagocytic index, K: condition factor

### 3.2 Expression of antioxidant and immune response genes

**Fig 1:**
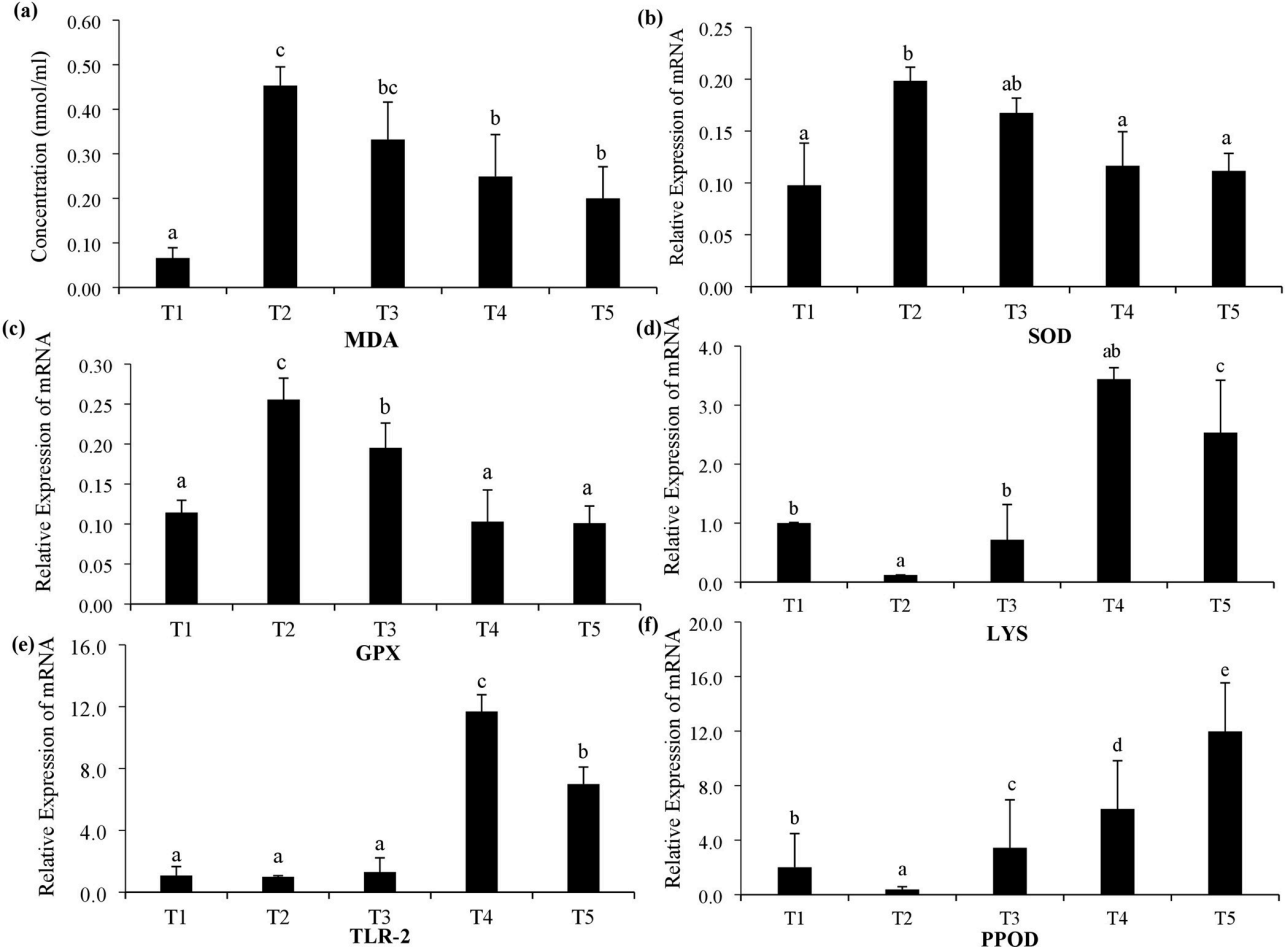
Concentration of malondialdehyde (MDA) in hemolymph measured by ELISA (a). Relative expression of mRNA of superoxide dismutase (SOD) (b), lysozyme (LYS) (c), glutathione peroxidase (GPX) (d), toll like receptor-2 (TLR-2) (e) and prophenolperoxidase (PPOD) (f) in four different treatments (T1, lows stocking density, T2, high stocking density, T3: lauric acid, T4: N-acetylcysteine, T5: lauric acid+N-acetylcysteine.

The levels of MDA were significantly different (p<0.05) between all treatments (Fig 1A), however, T4 and T5 were found to be in same subset. Concentration of MDA were noted to lower in treatments fed with NAC (T4 and T5) supplementation as compared to HSD control. Profile of all antioxidant and immune response genes showed significant difference (p<0.05) between all five treatments (Fig 1B–1F). The levels of SOD were significantly (p<0.05) lower in treatments fed with NAC and their combination (T4 and T5) (Fig 1B) as compared to T2. Similar results were observed in the profile of GPX when shrimp fed with NAC supplemented diet showed significantly (p<0.05) lowest levels (Fig 1C). Enhanced expression of LYS was noted in dietary supplementation treatment of NAC (T4) and NAC+LA (T5) (Fig 1D). The highest expression of TLR-2 was observed in shrimp fed with NAC supplemented diet (T4) and combination of NAC and LA (T5) (Fig 1E). A gradual increase in expression of PPOD was noted in T3, T4 and T5 (Fig 1F). The highest expression of this gene was shown in shrimp fed with combination of NAC and LA. In LSD and HSD treatments which were not given any supplement showed lower expression of all genes as compared to other dietary treatments.

## 4. Discussion

The present study demonstrated that dietary supplementation of NAC (0.2%) and LA (0.2%) can improve growth, antioxidant and antimicrobial capacity in Pacific white shrimp. Research on the impact of N-acetyl-L-cysteine (NAC) or lauric acid supplementation on the growth and innate immunity of shrimp is currently limited. Consequently, there is a scarcity of published data specifically addressing and discussing these effects in relation to this species. However, studies reported on humans, broiler, pig and fish strongly support that NAC plays critical role as ROS scavenger/antioxidant and protects from damage caused by lipid peroxidation, DNA damage, oxidation of proteins and apoptosis [12, 18–23, 25].

Findings of present study show that higher levels of SOD and GPX in shrimp exposed to stress caused by high stocking density are indicator of active ROS homeostatic mechanism [31, 32]. NAC might have enhanced the intracellular cysteine pool; released protein thiols through disulfide cleavage, increased glutathione levels; and enhanced the activity of glutathione-dependent detoxification of H_2_O_2_ by glutathione peroxidase as indicated by its high concentrations in T4 and T5 [12, 33, 34]. The presence of L-cysteine is a constraining factor in the biosynthesis of glutathione, and NAC is employed as a precursor to L-cysteine. However, the mechanism through which NAC stimulates glutathione synthesis and its in vivo transport is yet to be determined [18]. The enhanced activity of SOD dependent dismutation of superoxide anion (O_2_• -) into H_2_O_2_ and O_2_ might also be due to direct (5) or indirect [18, 22] actions of NAC as observed in mice [35]. This finding is further justified by lower concentrations of MDA in NAC fed treatments which shows that SOD and GPX acted as termination products for lipid peroxidation [16, 21].

The Prophenoloxidase (PPOD) activation system plays a crucial role in pathogen recognition and defense in crustaceans by pattern recognition proteins (PRP) and PAMP in hemocytes [36]. Detection of pathogen invasion in shrimps activates PPOD to undergo hydrolysis to form phenoloxidase which in turn, triggers the production of melanin [37]. Melanin and specific intermediates produced during this process demonstrate potent bactericidal activity, effectively eliminating pathogens and promoting wound healing [37]. Similarly, lysozyme is an enzyme that exerts its activity by hydrolyzing the -1,4-glycosidic bonds between N-acetylmuramic acid and N-acetylglucosamide in the polysaccharide backbone of the peptidoglycans of the gram-positive bacterial cell wall [28]. Given its ability to disrupt the bacterial cell wall, lysozyme is recognized as an endogenous antibiotic, playing a vital role in the innate defense against microbes. Our findings reveal an upregulation of LYS and PPOD expression, number of hemocytes and phagocytic index in shrimp fed with NAC and LA supplemented diets, indicating an improved immune function enhanced by these two compounds when compared with controls.

Lauric acid is a naturally occurring fatty acid with significant antibacterial activity against a wide range of bacteria. It induces microbial membrane disruption, activates reactive oxygen species production in pathogens, magnify the outgrowth inhibition effect [5–7]. This medium chain fatty acid induces apoptosis mediated through the phosphorylation of epidermal growth factor receptor and inhibition of thymidylate synthase expression in toxic cells [38]. Due to its surfactant properties, LA causes permeabilization of the cytoplasmic membrane in certain gram-positive bacterial cells and release of low-molecular-mass proteins (<20 kDa) from cells [39]. These antimicrobial properties of LA supplementation to improve immune resistance in present study were monitored by the levels of TLR-2. Lauric acid triggers the dimerization of Toll-like receptor-2 (TLR-2) with either TLR-1 or TLR-6, resulting in the activation of MyD88-dependent signaling pathways (19). This activation leads to the subsequent production of proinflammatory cytokines, including IL-12, IL-6, and TNF-α, along with chemokines and their receptors [10] thereby establishing long-term protective immunity. In present study, profile of TLR-2 increased in treatments fed with supplementation of LA as compared to controls. This finding confirms the potential use of LA as an additive in shrimp feed to enhance immunity against bacteria and viruses [40–42]. However, further studies are required to investigate its role in TLR mediated signaling pathways against common and virulent pathogens in aquaculture.

Similar to improved antioxidant and antimicrobial activity, better growth in NAC and LA fed treatments may also be attributed to the multifaceted mechanisms of these nutraceuticals, to activate anti-inflammatory and immunomodulatory functions as observed in common carp [19], Chinese mitten crabs [24] and Nile tilapia [20]. Higher levels of total protein and glucose in T3 and T5 also indicate the improvement in nutrient supply and their transport via globulin and albumin, which may be due to inclusion of LA in diets as observed in poultry [11] and black seabream [9]. Similar effects of NAC and LA on growth have been previously indicated by better gut microbiota [20], longer intestinal villi [24] and improved regulation of microbiome, thereby enhancing gut health [30]. Particularly, LA maintains its stability as it traverses the gastrointestinal tract, ensuring its eventual absorption [43]. This characteristic brings LA into direct interaction with gut microbiota, contributing to the enhancement of host health and physiology through improved metabolism and immunity. Higher levels of glucose observed in T1 as compared to that in T2 could be attributed to fast metabolism of available glucose to cope with high energy needs (Xu et al., 2018).

## 5. Conclusion

The findings of the present study confirm that the supplementation of NAC and LA (0.2%) in the diet of Pacific white shrimp can enhance growth, antioxidant, and immune responses. Further investigation is required to determine the optimal doses at higher stocking densities that can be more effective against common aquaculture pathogens. Unfortunately, due to restrictions on the use of pathogenic bacteria in laboratory, present study could not subject the treatments to challenge with virulent bacteria. Future studies can explore these aspects, which will be beneficial in enhancing the antioxidant and immune responses of Pacific white shrimp in intensive culture.

## Supporting information

S1 Data (XLSX)
